# Assessing gender role attributes in native Persian speakers: translation, cultural adaptation, and validation of the Persian version of the personal attribute questionnaire

**DOI:** 10.3389/fsoc.2025.1535815

**Published:** 2025-03-28

**Authors:** Golnoosh Kamyab, Marie-Pierre Gagnon-Girouard, Zoha Deldar, Ladan Ghazi-Saidi, Mathieu Piché

**Affiliations:** ^1^Department of Anatomy, Université du Québec à Trois-Rivières, Trois-Rivières, QC, Canada; ^2^Department of Psychology, Université du Québec à Trois-Rivières, Trois-Rivières, QC, Canada; ^3^Department of Communication Disorders, University of Nebraska at Kearney, Kearney, NE, United States

**Keywords:** expressivity, instrumentality, femininity, masculinity, androgyny, sex, age

## Abstract

The Personal Attributes Questionnaire (PAQ) assesses gender roles, including expressivity (femininity) and instrumentality (masculinity), which reflect socially and culturally defined feminine and masculine ways of thinking, feeling, and behaving. The PAQ allows the assessment of gendered traits, beyond the traditional binary view. With the inclusion of gender-related factors in various research fields, the PAQ has been validated in multiple languages and cultures, including German, Chinese, and French. However, a Persian version has not yet been validated. This study aims to validate and examine the test–retest reliability of the culturally adapted Persian PAQ. A total of 436 native Persian speakers (302 females, 134 males) completed the questionnaire. Exploratory and confirmatory factor analyses were conducted to evaluate the factorial structure and validity of the Persian PAQ. In addition, test–retest reliability was assessed to ensure its consistency over time. Exploratory factor analysis confirmed a two-factor structure, although ‘Active’ loaded on both factors. The results showed a good fit (RMSEA = 0.070, GFI = 0.91 and AGFI = 0.88), acceptable internal consistency (expressivity: α = 0.70, instrumentality: α = 0.72), and moderate to excellent test–retest reliability for instrumentality (ICC = 0.92) and expressivity (ICC = 0.69). The results indicate that women and younger adults were more likely to show lower expressivity and instrumentality (undifferentiated), or higher expressivity and instrumentality (androgynous) compared with males and older adults, respectively. These findings support the validity and reliability of the Persian PAQ and show that gender role attributes are influenced by sex and age. The Persian PAQ will enable to consider the influence of gender in health, sociology, and psychology research.

## Introduction

With the recognition of sex and gender as critical factors in health research, recent guidelines strongly recommend their inclusion in research designs and data analyses to enhance the accuracy and representativeness of study findings across diverse populations (Canadian Institutes of Health Research, 2023). Since gender is a social construct shaped by different cultures and societies, there is a growing need for valid and culturally adapted measures to assess gender-related attributes across diverse contexts.

The Canadian Institutes of Health Research (CIHR) define sex and gender as distinct concepts that play a crucial role in the understanding of human identity and behavior. Sex is a biological attribute of males and females determined at birth based on the reproductive system. Gender is used to describe the socially constructed roles, identity expressions, and behaviors associated with men, women, and gender-diverse individuals (Canadian Institutes of Health Research, 2023). Sex can be self-reported, but gender is more challenging to assess because it is constructed through complex social interactions (Archer, 1989).

Gender roles and stereotypes are produced by the interactions between individuals and their environment. They are identified as feminine or masculine through daily activities, behaviors, and emotions (Goffman, 1977; Bhatia and Bhatia, 2021). Thus, individuals identify and adopt behaviors and traits considered appropriate for their gender from early childhood (Kohlberg, 1966; Spence, 1984). Traditionally, gender roles were based on men being the primary providers for their families and making important family decisions (Bakan, 1966; Brody, 1999; Fard et al., 2021), while women were taking care of children (Eagly and Steffen, 1984). Expressive attributes such as showing emotions and nurturing were associated with the roles of women, while instrumental attributes like independence, assertiveness, and task focus were associated with the roles of men (Chaplin, 2015; Emde and Hewitt, 2001; Erikson, 1964). However, social roles continuously evolve and vary across cultures. Thus, tools used to assess gender role attributes must be adapted and validated for specific populations.

Gender role attributes have evolved from a unidimensional theory to the bidimensional model (Bem, 1974; Spence et al., 1975). In this contemporary model, masculinity and femininity are two independent dimensions. However, individuals from both sexes can adopt a combination of gender roles associated with both masculinity and femininity, thereby transcending traditional gender roles (Spence and Helmreich, 1978). Accordingly, instrumentality is also valued in women and expressivity is also valued in men (Swazina et al., 2004; Ebert et al., 2014).

Gender roles are context dependent. They differ across social groups, socioeconomic levels, regional or national identities, and they evolve with cultural transformations (Bhatia and Bhatia, 2021; van de Vijver, 2007). For example, Canadian women exhibit greater autonomy, make more decisions and actively change their environment compared with Iranian women, which typically tend to adapt to their environment (Tavakoli, 2012). It was also suggested that the recent cultural transformations in Iran have increased women’s engagement in the public sphere, leading to a decrease in the desirability of their traditional roles (Rafatjah, 2012; Sohrabzadeh et al., 2020). In addition to the fact that gender roles vary across countries and cultures, sociodemographic factors such as age, education, and employment status also play a crucial role in shaping gender role development (Calvo-Salguero et al., 2008; Hofstede et al., 2010; Lynott and McCandless, 2000; Huyck, 1999). For example, younger individuals tend to be more sex-typed, with women displaying more feminine traits and men exhibiting more masculine traits. However, as people age, the differences between men and women gradually decrease, leading to a greater tendency toward androgyny (Feldman et al., 1981; Gutmann, 1977; Hofstede et al., 2010).

Historically, gender roles in Iran were primarily shaped by family structures with minimal state intervention (Malekan, 2015). However, in the past fifty years, Iranian women have been placed in a contradictory position, expected to balance cultural and religious expectations with evolving societal demands; women are exposed to societal pressure to be traditional, devoted wives and mothers, while also being modern, educated, and active in the socio-political life (Malekan, 2015). On the other hand, masculinity among Iranian men has evolved from a rigid, state-endorsed patriarchal model, to a more fluid, ambivalent construct among younger men, influenced by globalization, economic challenges, and changing social norms (Balslev, 2019). In a study using the Bem Sex Role Inventory (BSRI) among Iranian adults, approximately 46% of men and 56% of women were classified as cross-sexed (Undifferentiated and androgynous) rather than sex-typed (Masculine and Feminine) (Alavi et al., 2014). This finding suggests a shift in Iranian society from traditional gender roles toward emerging, more flexible gender role identities.

The current tool used to measure expressivity and instrumentality is the short form of the Personal Attributes Questionnaire (PAQ), developed by Spence, Helmreich, and Stapp in 1978. The PAQ comprises twenty-four bipolar questions, each describing self-reported characteristics that are stereotypically associated with masculinity (8 questions) or femininity (8 questions). The three scales include Masculinity (M), Femininity (F), and Masculinity-Femininity (M-F). The Masculinity scale comprises traits considered socially desirable for both sexes, but more frequently observed in men, while the Femininity scale contains traits considered desirable for both sexes, but more frequently observed in women. The M-F (Masculinity-Femininity) scale consists of characteristics whose social desirability varies between the two sexes according to traditional gender norms. For example, aggressiveness is often considered a desirable trait for males, while non-aggressiveness is traditionally seen as desirable for females (Basow and Rubenfeld, 2003; Tannen, 1990).

The content of the questionnaire aligns with the bidimensional model of gender roles, where instrumentality traits are associated with masculinity, and expressivity traits are associated with femininity (Spence and Helmreich, 1978). Recent research using the PAQ has relabeled the original scales, referring to “instrumentality” instead of masculinity, and “expressivity” instead of femininity. Although these two factors are slightly correlated, they are considered independent (Spence and Helmreich, 1978).

Based on PAQ scores, individuals are categorized as sex-typed or cross-sex categories. Sex-typed individuals (*Masculine* and *Feminine*) align with traditional gender roles, for which masculinity is greater in men and femininity is greater in women. In contrast, cross-sex categories do not align with traditional gender roles and comprise *androgynous* individuals characterized by high levels of both masculinity and femininity, and *Undifferentiated* individuals characterized by low levels of both masculinity and femininity (Spence and Helmreich, 1978).

Since its development, the PAQ has been used in the fields of health, psychology, and sociology (Mayor, 2015; Rosen et al., 2000; Shifren and Bauserman, 1996; Thorn et al., 2004; Tibubos et al., 2022; Zentner et al., 2023). For example, in a mental health study, Tibubos et al. (2022) found that higher levels of both femininity and masculinity were associated with lower distress in the German population. Similarly, in pain research, the PAQ has been used to explore the influence of gender roles on pain perception and responses (Dixon et al., 2004). Considering the importance of this questionnaire in gender and health research, the fact that gender roles are shaped by cultural and societal norms, as well as the current lack of a culturally adapted instrument for the Iranian context, this study aims to validate the Expressivity (Femininity) and Instrumentality (Masculinity) subscales of the short-form PAQ among native Persian speakers in Iran. Validating this questionnaire in the Iranian context would support its cross-cultural applicability and ensure that gender-related attributes can be assessed reliably in Iranian individuals. Specifically, we seek to determine whether the Persian PAQ aligns with the established instrumentality and expressivity dimensions. We hypothesize that: (1) The Persian PAQ will exhibit a two-factor structure corresponding to instrumentality and expressivity, (2) The two factors will be independent, as indicated by a low between-factor correlation, (3) All instrumentality-related items will primarily load onto the instrumentality factor, and (4) All expressivity-related items will primarily load onto the expressivity factor.

This study on the Persian version of the PAQ includes the confirmation of its construct validity through exploratory and confirmatory factorial analyses, the assessment of internal consistency, and the assessment of test–retest reliability in a subsample of the study. The results are anticipated to address existing gaps in gender assessment tools in the Persian language and culture. Due to cultural differences between Iran and Western countries, the use of the Persian PAQ in future research could provide insights into gender roles evolution in Iran and allow for comparisons with other countries and cultures. Since sex and gender are often used interchangeably in the Iranian society and only two gender identities are officially recognized, alternative tools are needed to include gender in research designs and data analyses. The Persian adaptation of the PAQ, which measures gender roles within the Iranian cultural context, could help advance research in several fields, including health and gender studies. In addition, this study examines the influence of age, education, and employment status on gender classification to provide a deeper understanding of how these factors contribute to gender role development in Iran. Moreover, the results will offer a broader perspective on future transcultural studies using the PAQ in various populations and languages.

## Materials and methods

### Ethics approval

This study received approval from the Research Ethics Board of Université du Québec à Trois-Rivières and was conducted in accordance with the Declaration of Helsinki. Informed consent was provided electronically by participants before accessing the online questionnaire. Participants were informed that data collection was confidential and that they were free to withdraw from the study at any time during the questionnaire completion without prejudice. No compensation was offered to participants.

### Participants

The sample size of the study is based on a respondent-to-item ratio of 15:1 (Pedhazur and Kerlinger, 1982). The recruitment was conducted through social media advertisements on platforms such as WhatsApp, Instagram, Telegram, and Twitter. To be eligible for participation, individuals had to be over 18 years old and proficient in reading and writing Persian as their native language. A total of 659 native Persian speakers aged between 18 and 76 years completed the online questionnaire. The final sample included 436 participants (302 females; 134 males), who provided responses to all questions. The remaining participants were excluded for incomplete questionnaires or invalid answers as determined with a trap question. Table 1 shows the sociodemographic characteristics of the 436 participants.

**Table 1:** Sociodemographic characteristics of participants.

Baseline characteristic	Male		Female		t/x^2^	df	p *
	*n*	*%*	*n*	*%*			
Sex (*n* = 436)	134	30.7	302	69.2			
Classification					18.3	3	<0.001
Feminine	15	11.1	76	25.1			
Masculine	36	26.9	42	13.9			
Androgynous	51	38.1	102	33.8			
Undifferentiated	32	23.9	82	27.2			
Age (Mean ± SD)	35.5 ± 10.1		34.6 ± 10.4		0.9	434	0.4
Marital status					7.7	3	0.052
Never Married	67	50	115	38.1			
Married	56	41.8	152	50.3			
Divorce	5	3.7	25	8.3			
Other	6	4.5	10	3.3			
Education level					0.6	2	0.74
Completed high school	20	14.9	54	17.9			
College or university degree	68	50.7	146	48.3			
Graduate or professional degree	46	34.3	102	33.8			
Employment status					31.1	2	<0.001
Employed	112	83.6	172	57			
Unemployed	14	10.4	106	35.1			
Study	8	6	24	7.9			
Ethnicity					7.9	5	0.16
Fars	78	58.2	174	57.6			
Turk	39	29.1	61	20.2			
Kurd	5	3.7	20	6.6			
Lurs	4	3	18	6			
Gilak-Mazan	4	3	18	6			
Other	4	3	11	3.6			
Religion					21.0	2	<0.001
Islam	55	41	194	64.2			
Atheist	69	51.5	90	29.8			
Other	10	7.5	18	6			
Income					53.4	4	<0.001
Low	61	45.5	188	62.3			
Middle	38	28.4	27	8.9			
Upper middle	8	6	5	1.7			
High	20	14.9	21	7			
Other	7	7	61	20.2			
Society					1.84	1	0.18
Rural	2	1.5	12	4			
Urban	132	98.5	290	96			

*N = 436.*

### General procedures

After confirming their eligibility as part of the questionnaire and providing informed consent, participants filled a sociodemographic questionnaire, followed by the short form of the PAQ in Persian language (Qualtrics, Provo, UT). A trap question (attention check – “What is the second letter of the alphabet?”) was placed immediately after completing the PAQ to ensure that questions were read and answered correctly. To assess the reliability of the PAQ, the questionnaire was filled one month later (Shultz and Whitney, 2005) by 44 participants who provided their email address and accepted to participate a second time upon invitation.

### Adaptation of the personal attribute questionnaire from English to Persian

The PAQ was translated by two Persian native speakers. The Persian version was then back-translated by a bilingual translator and a researcher experienced in translating psychological scales and questionnaires (Guillemin et al., 1993). The research team members discussed the back-translation to improve the Persian version that was submitted to 20 native Persian individuals from the general population including male and female adults of different ages. This led to the final Persian version of the PAQ produced by the research team (Beaton et al., 2007). A modification was implemented for clarity, accounting for the distinct linguistic characteristics of English and Persian. To identify appropriate letters for the 5-point Likert scale in the Persian version, the Persian “abjad” sequence, which is the native script order in Persian, was selected instead of the English alphabet to better align with the Persian linguistic context.

### Personal attributes questionnaire

The short form of the PAQ comprises two subscales, “F” (femininity or expressivity) and “M” (masculinity or instrumentality), designed to assess gender role attributes. Within each subscale, 8 bipolar items capture gender stereotypes related to expressivity and instrumentality. The F subscale consists of expressivity items, such as emotional, devoted to others, gentle, helpful to others, kind, aware of others’ feelings, understanding, and warm. The M subscale consists of instrumentality items, including independence, active, competitive, decisive, never gives up, stands up to pressure, self-confident, and feel superior. The M and F subscales are statistically independent and uncorrelated in both sexes. The Cronbach’s alpha coefficients in the original validation study were 0.82 for expressivity (F) and 0.85 for instrumentality (M), demonstrating internal consistency.

Each item was rated using a 5-point Likert scale, ranging from 0 (“Not at all”) to 4 (“Very”). The score for each subscale was computed by adding the scores of the 8 items. The M and F subscale are scored to obtain higher scores for higher masculinity and femininity, respectively (Spence et al., 1974). The PAQ includes an M-F subscale comprising eight items, based on the assumption that masculinity and femininity are opposites, and that social desirability varies between the two sexes. Two items refer to instrumental traits, and six items relate to emotional vulnerability and the need for emotional support. In this study, the M-F subscale was not included for several reasons. Firstly, research has shown that this subscale presents conceptual and psychometric issues and exhibits the lowest reliability due to its mixed content (Hill et al., 2000). Secondly, while the Masculinity (M) and Femininity (F) subscales are widely reported and well-defined in the literature, the interpretation of the M-F subscale remains unclear (McCreary and Steinberg, 1992). Thirdly, the M-F subscale differs conceptually from the M and F subscales (McCreary and Steinberg, 1992). Indeed, modern gender-role research treats instrumentality and expressivity as independent constructs, meaning that an individual can score high on both traits. However, the M-F subscale conceptualizes them as bipolar, suggesting that higher instrumentality implies lower expressivity and vice versa, which is inconsistent with contemporary gender theories. Lastly, to keep the questionnaire concise, this subscale was excluded from the present study (McCreary and Steinberg, 1992).

### Statistical analysis

Factorial analyses were conducted using Factor software (Version 12.4.5.0). An exploratory factorial analysis was conducted on the scores of the 16 items to assess latent factors. Subsequently, a confirmatory factor analysis (CFA) was performed out using Amos (Version 26) to confirm the plausibility of the factor structure within the Persian population and its alignment with the theoretical framework (Brown, 2015).

For the exploratory factorial analysis, sample adequacy and suitability were assessed using the Kaiser-Meyer-Olkin (KMO) test and the Bartlett test of sphericity. Factors were extracted through parallel analysis when eigenvalues exceeded 1. Given the asymmetrical distribution and ordinal nature of the data, the Robust Diagonally Weighted Least Squares (RDWLS) estimation method was used with the oblique Promin rotation. A minimum factor loading of 0.30 was required to demonstrate the association between an item and a factor.

In the confirmatory factor analysis, the factorial structure was evaluated using goodness-of-fit statistics, including the Root Mean Square Error of Approximation (RMSEA), Adjusted Goodness of Fit Index (AGFI), and Goodness of Fit Index (GFI). Internal consistency was measured using Cronbach’s alpha (Dornyei, 2007).

To assess test–retest reliability, Intraclass Correlation Coefficient (ICC) estimates with 95% confidence intervals were calculated for the F and M subscales. This analysis was conducted in IBM SPSS Statistics (Version 29) using a two-way mixed-effects model and absolute agreement.

## Results

### Sample characteristics

Sample characteristics are presented in Table 1. No significant difference was observed between males and females for age, marital status, education, and ethnicity (all *p*’s > 0.05). However, significant differences were observed between males and females for gender, the employment status, religion, and income (all *p*’s < 0.05; see Table 1 for details).

To compare the mean scores for masculinity-related and femininity-related items between females and males, two separate MANOVAs were conducted using the 8 items of each subscale as dependent variables and sex as a between subject factors (see Table 2 for details). As expected, the femininity score was significantly greater in females compared with males (23.2 vs. 22.0, respectively, F_8, 427_ = 6.3, *p* < 0.001, η_p_^2^ = 0.11), while the masculinity score was significantly greater in males compared with females (19.7 vs. 18.1, respectively, F_8, 427_ = 3.1, *p* = 0.002, η_p_^2^ = 0.055). Planned comparisons revealed that females scored higher than males on femininity-related items (*p*’s < 0.05), except for *‘Gentle’* and *‘Helpful to others’*, for which the score was not significantly different between sexes (*p* = 0.15 and *p* = 0.56, respectively), while males scored higher on masculinity-related items compared with females (*p*’s < 0.05), except for ‘Active’, for which the score was not significantly different between sexes (*p* = 0.79).

**Table 2:** Mean and standard deviation for each item of the personal attribute questionnaire.

Items	Total		Male		Female	
	x̄	SD	x̄	SD	x̄	SD
Factor 1: masculinity-instrumentality (M)						
Independent	2.71	1.20	2.94	1.07	2.61	1.24
Active	2.65	1.06	*2.63*	*1.02*	*2.66*	*1.08*
Competitive	1.95	1.17	2.14	1.24	1.87	1.13
Can make decisions easily	2.21	1.20	2.28	1.13	2.17	1.23
Never gives up	1.94	1.12	1.98	1.07	1.92	1.15
Self-confident	2.32	1.07	2.50	0.97	2.24	1.10
Feels superior	2.32	0.93	2.54	0.85	2.23	0.95
Stands up well under pressure	2.48	1.14	2.68	1.02	2.39	1.17
Factor 2: femininity-expressivity (F)						
Emotional	2.88	0.97	2.73	0.99	2.95	0.96
Able to devote self completely to others	2.37	1.16	2.11	1.12	2.48	1.16
Gentle	2.58	0.95	2.68	0.91	2.54	0.97
Helpful to others	3.11	0.88	3.15	0.85	3.10	0.89
Kind	3.33	0.80	3.32	0.81	3.33	0.79
Aware of feelings of others	2.81	0.90	2.51	1	2.94	0.82
Understanding of others	3.01	0.86	2.80	1	3.10	0.77
Warm in relation with others	2.72	1.02	2.69	1.01	2.74	1.03

Response scale: 0 (“Not at all”) to 4 (“Very”). *N* = 436; *n* (male) = 134; *n* (female) = 302.

### Factorial analyses

#### Exploratory factorial analysis

The sample size was adequate, with KMO values of 0.81 for the whole sample, 0.80 for females, and 0.77 for males. The covariance among items was appropriate for factorial analysis, as indicated by the Bartlett test of sphericity: χ^2^ (120, *N* = 436) = 1768.6, *p* < 0.001 for the whole sample; χ^2^ (120, *N* = 302) = 1299.7, *p* < 0.001 for females; and χ^2^ (120, *N* = 134) = 712.2, *p* < 0.001 for males. The exploratory factor analysis identified three eigenvalues greater than 1 (4.1, 2.4, and 1.3) for the whole sample. Four eigenvalues exceeded 1 for females (4.1, 2.3, 1.3, and 1.1) and males (4.5, 2.4, 1.3, and 1.0) separately. However, the parallel analysis indicated that only two factors exceeded the 95^th^ percentile for all samples. Together, these two factors accounted for 45.3% of the total variance in the whole sample (*λ* = 3.5, 28.4% and λ = 1.8, 16.9%, respectively). Similarly, these factors accounted for 43% of the total variance for females (λ = 3.6, 27.8% and λ = 1.7, 15.2%, respectively). For males, the two factors explained 49.1% of the total variance (λ = 3.9, 32% and λ = 1.8, 17.1%, respectively).

**Figure 1:**
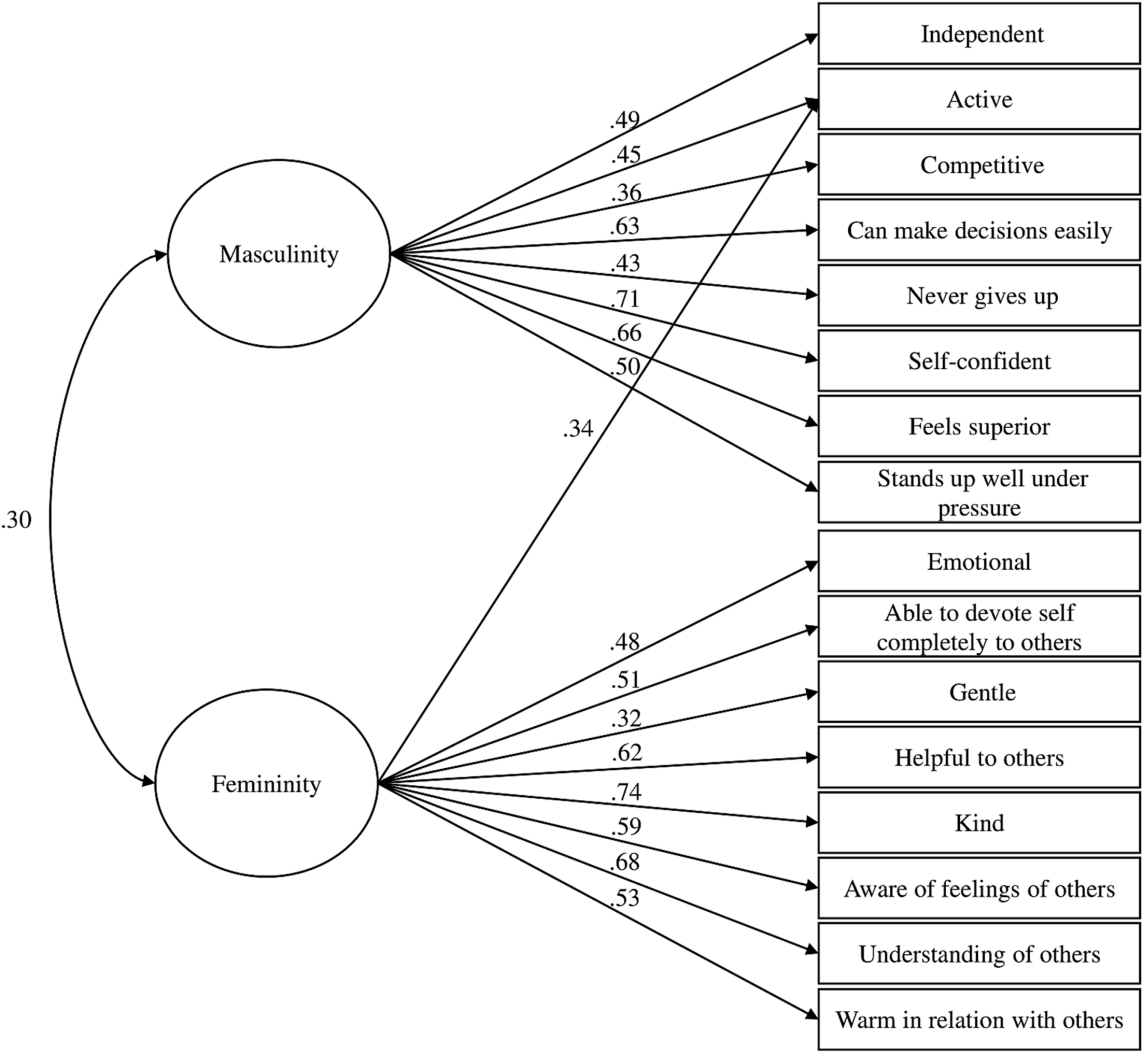
Two-factor model of the personal attributes questionnaire with standardized coefficients. All items predominantly load on their respective factors, except for the item “active,” which loads on both factors.

Table 3 shows the factor loadings of the 16 PAQ items of interest. Masculinity items loaded more on one factor and all Femininity items loaded more on the other factor. The range of loadings on the intended factor was 0.36–0.71 for masculinity and 0.32–0.74 for femininity for the whole sample (Figure 1). All loadings on the unintended factor were weak, except for ‘Active,’ which showed loadings greater than the satisfactory standard of 0.3 for both factors (0.34 and 0.45) (Costello and Osborne, 2005). In the female and male samples separately, all masculinity-related items (with loadings ranging from 0.32 to 0.72 for females and 0.35 to 0.68 for males) loaded significantly on one factor, whereas femininity-related items primarily loaded on the other factor (with loadings from 0.26 to 0.80 for females and 0.45 to 0.80 for males). However, the item ‘Gentle’ did not load significantly on either factor for females, and ‘Active’ loaded on both factors for males. Promin rotation (kappa = 4) revealed a weak correlation between the two factors: 0.30 for the whole sample, 0.24 for males, and 0.36 for females (Lorenzo-Seva and Ferrando, 2019).

**Table 3:** Results from the exploratory factorial analysis.

PAQ items	Factor loading					
	Total (436)		Male (134)		Female (302)	
	F1	F2	F1	F2	F1	F2
Factor 1: masculinity-instrumentality (M)						
Independent	0.09	**0.49**	0.13	**0.36**	0.16	**0.49**
Active	**0.34**	**0.45**	**0.55**	**0.35**	0.29	**0.44**
Competitive	−0.13	**0.36**	0.04	**0.41**	−0.13	**0.32**
Can make decisions easily	−0.13	**0.63**	0.15	**0.68**	−0.20	**0.65**
Never gives up	−0.01	**0.43**	0.19	**0.41**	−0.04	**0.43**
Self-confident	−0.01	**0.71**	0.01	**0.66**	0.05	**0.72**
Feels superior	−0.04	**0.66**	0.13	**0.58**	−0.01	**0.66**
Stands up well under pressure	0.04	**0.50**	0.16	**0.45**	0.06	**0.49**
Factor 2: femininity-expressivity (F)						
Emotional	**0.48**	−0.26	**0.48**	−0.35	**0.50**	−0.24
Able to devote self completely to others	**0.51**	−0.21	**0.45**	−0.18	**0.54**	−0.25
Gentle	**0.32**	0.05	**0.55**	−0.19	0.26	0.10
Helpful to others	**0.62**	0.03	**0.77**	−0.15	**0.61**	0.00
Kind	**0.74**	0.00	**0.80**	−0.09	**0.80**	−0.07
Aware of feelings of others	**0.59**	0.06	**0.51**	0.15	**0.62**	0.03
Understanding of others	**0.68**	0.09	**0.73**	0.12	**0.66**	0.06
Warm in relation with others	**0.53**	0.20	**0.61**	0.08	**0.52**	0.20

F = Factor. The extraction method was Diagonally Weighted Least Squares (RDWLS) with an oblique (Promin) rotation. Factor loadings above than 0.30 are in bold. KMO = 0.80; KMO male = 0.77; KMO female = 0.80.

#### Confirmatory factorial analysis

Model Fit Statistics for the confirmatory factor analysis are detailed in Table 4. The Root Mean Square Error of Approximation (RMSEA) indicated an acceptable fit, with values of 0.070 (90% CI: 0.061–0.079) for the whole sample, 0.070 (90% CI: 0.059–0.081) for females, and 0.075 (90% CI: 0.056–0.093) for males. In addition, both the Goodness-of-Fit Index (GFI) and the Adjusted Goodness-of-Fit Index (AGFI) showed acceptable to excellent fit, achieving 0.91 and 0.88 for the whole sample, 0.90 and 0.87 for females, and 0.86 and 0.81 for males. Thus, the model is deemed adequate and provides a good fit to the data (Yu, 2002). Figure 1 displays the results of the CFA with standardized parameters.

**Table 4:** Model fit statistic.

Fit indices	χ^2^	df	RMSEA (CI)	GFI	AGFI
Two-factor model (whole sample)	321.5*	103	0.070 (0.061–0.079)	0.91	0.88
Two-factor model (females)	254.5*	103	0.070 (0.059–0.081)	0.90	0.89
Two-factor model (males)	179.2*	103	0.075(0.056–0.093)	0.86	0.81

χ2 = chi-square test: *p < 0.001; RMSEA = Root Mean Square Error of Approximation; GFI = Goodness-of-Fit index; AGFI indicates Adjusted Goodness-of-Fit Index.

In order to examine if the model could be improved by accounting for the loading of item ‘Active’ on the femininity factor, we conducted a second confirmatory factor analysis with this additional covariance. The Root Mean Square Error of Approximation (RMSEA) for this modified model indicated a slightly better fit, with values of 0.063 (90% CI: 0.055–0.072) for the whole sample, 0.067 (90% CI: 0.056–0.078) for females, and 0.061 (90% CI: 0.040–0.081) for males. Besides, both the Goodness-of-Fit Index (GFI) and the Adjusted Goodness-of-Fit Index (AGFI) showed good to excellent fit, achieving 0.92 and 0.89 for the whole sample, 0.91 and 0.87 for females, and 0.88 and 0.83 for males, respectively. Thus, the modified model is also deemed adequate and provides a good fit to the data (Yu, 2002).

#### Internal consistency of the Persian version of the personal attribute questionnaire

The psychometric properties were evaluated using the Cronbach’s alpha coefficient. The Femininity and Masculinity factors yielded acceptable Cronbach’s alpha coefficients (whole sample: α = 0.70 and 0.72; Females: α = 0.69 and 0.72; Males: α = 0.73 and 0.79, respectively) (Dornyei, 2007). To examine if the removal of the item ‘Active’ from the masculinity scale could improve consistency, we computed the Cronbach’s alpha coefficient for the Masculinity subscale with 7 items. The Cronbach’s alpha coefficient was reduced to 0.69, indicating that the 8-item subscale is preferable. These findings underscore the internal consistency of the Persian version of the Personal Attributes Questionnaire for the whole sample, but also for the females and males taken separately.

#### Test–retest reliability of the Persian version of the personal attribute questionnaire

Test–retest reliability was assessed with data from 44 participants who answered the questionnaire twice with a one-month interval. Statistics are presented in Table 5. The masculinity items yielded an ICC of 0.92 (95% CI, 0.85–0.96), which indicates excellent test–retest reliability. For femininity, the ICC was 0.69 (95% CI, 0.42–0.82), indicating moderate reliability (Koo and Li, 2016).

**Table 5:** Test–retest reliability.

Factor	ICC	df	CI (95%)
Factor 1 (F)	0.69	43	0.42–0.82
Factor 2 (M)	0.92	43	0.85–0.96

ICC = Intraclass Correlation Coefficient; CI = Confidence Interval.

#### Gender classification

The gender classification was performed using the median split method of scores (Spence and Helmreich, 1978). The classification includes 4 gender categories based on whether the scores for masculinity and femininity fall below or above the median values. This classification includes *Androgynous* (above the median for both Femininity and Masculinity), *Feminine* (above the median for Femininity and below the median for Masculinity), *Masculine* (above the median for masculinity and below the median for femininity), and *Undifferentiated* (below the median for both Femininity and Masculinity). Due to the unequal number of females and males in the sample, the medians for the femininity and masculinity subscales were calculated separately for females and males. The mean of these medians was then calculated for masculinity and femininity and was used for classification (Spence and Helmreich, 1978). On a maximum of 32 for each subscale, the median values were 23 for Femininity and 18 for Masculinity for females. For males, the median values were 22 for Femininity and 20 for Masculinity. Thus, the average medians used for classification was 22.5 for Femininity and 19 for Masculinity. Based on these scores, 11.1% of males were categorized as *Feminine*, 26.9% as *Masculine*, 38.1% as *Androgynous*, and 23.9% as *Undifferentiated*. Among females, 25.1% were categorized as *Feminine*, 13.9% as *Masculine*, 33.8% as *Androgynous*, and 27.2% as *Undifferentiated*. These results are detailed in Table 1.

##### Exploring sociodemographic influences on gender classification

Loglinear analyses were conducted to explore the influence of education, employment status, and age on gender classification, while accounting for sex.

#### Education

The differences between education (3 categories: Completed High School, College or University Degree, and Graduate or Professional Degree), gender (4 categories: Feminine, Masculine, Androgynous, and Undifferentiated) and sex (2 categories: Males, Females) were examined; the three-way loglinear analysis produced a final model that did not retain the three-way interaction (χ^2^(6) = 8.8, *p* = 0.19), but retained the two-way interactions (χ^2^(17) = 37.6, *p* = 0.003). Partial associations indicated that genders were different between sexes (χ^2^(3) = 18.3, *p* < 0.001), as expected, but not between education categories (χ^2^(6) = 10.3, *p* = 0.11). Moreover, Education categories were not significantly different between sexes (χ^2^(2) = 1.0, *p* = 0.61) (see Tables 6, 7).

**Table 6:** Interaction between sex and gender with education, employment, age for all gender classifications.

Interactions	Value	df	*p*
Sex*gender* education	8.8	6	0.19
Two-way interactions	37.6	17	0.003^a^
Sex* education	1.0	2	0.61
Gender* education	10.3	6	0.11
Gender*sex	18.3	3	< 0.001^b^
Sex*gender*employment	1.02	3	0.8
Two-way interactions	52.4	10	< 0.001^c^
Sex* employment	29.4	1	< 0.001^d^
Gender* employment	1.6	3	0.7
Gender*sex	15.4	10	< 0.001^e^
Sex*gender*age	14.5	12	0.27
Two-way interactions	57.1	31	0.003^f^
Sex* age	1.7	4	0.78
Gender*age	22.8	12	0.03^g^
Sex*gender	17.7	3	< 0.001^h^

^a, c, f significant interaction retained. b, d, e, h Significant differences between sexes. g Significant differences between genders.^

**Table 7:** Distribution of participants by education categories levels across four classifications.

Education level	Male				Female				Total			
	Undif	Fem	Masc	Andr	Undif	Fem	Masc	Andr	Undif	Fem	Masc	Andr
Completed high school	**3** 15.0	**1** 5.0	**5** 25.0	**11** 55.0	**19** 35.2	**9** 16.7	**5** 9.3	**21** 38.9	**22** 29.7	**10** 13.5	**10** 13.5	**32** 43.3
College or university degree	**17** 25.0	**5** 7.4	**17** 25.0	**29** 42.6	**37** 25.3	**43** 29.5	**21** 14.4	**45** 30.8	**54** 25.2	**48** 22.4	**38** 17.8	**74** 34.6
Graduate or Professional degree	**11** 23.9	**6** 13.0	**15** 32.6	**14** 30.4	**21** 20.6	**17** 16.7	**22** 21.6	**42** 41.2	**32** 21.6	**23** 15.5	**37** 25.1	**56** 37.8

Bold font numbers = *n*, normal numbers = %. Undif = undifferentiated; Fem = femininity; Masc = masculinity; Andr = androgynous.

For the Gender * Sex interaction, chi-square tests revealed that males were significantly more likely than females to be undifferentiated vs. feminine (OR = 1.98, *p* = 0.05) and masculine vs. feminine (OR = 4.43, *p* < 0.001), as expected. Males were also significantly less likely than females to be undifferentiated vs. masculine (OR = 0.46, *p* = 0.01) and feminine vs. androgynous (OR = 0.39, *p* = 0.004) (see Table 8).

**Table 8:** Loglinear analysis effects break down for gender * sex.

Classification	x^2^	*p*	Odds ratio
Male vs. female			
Undif vs. Masc	6.6	.01^a^	0.46
Undif vs. Fem	3.8	0.05 ^b^	1.98
Undif vs. Andr	0.85	0.4	0.78
Masc vs. Fem	17.5	<0.001 ^c^	4.34
Masc vs. Andr	3.6	0.057	1.71
Fem vs. Andr	8.2	0.004 ^d^	0.39

χ^2^ = Chi square. df = 1. ^a^ Males less likely than females to be Undifferentiated vs Masculine. ^b^Males more likely than females to be Undifferentiated vs Feminine. ^c^ Males more likely than females to be Masculine vs Feminine. ^d^ Males less likely than females to be feminine vs androgynous.

#### Employment

The differences between employment status (2 categories: Employed or Student, and Unemployed), genders (4) and sexes (2) were examined; the three-way loglinear analysis produced a final model that did not retain the three-way interaction (χ^2^(3) = 1.02, *p* = 0.8), but retained the two-way interactions (χ^2^(10) = 52.4, *p* < 0.001). Partial associations indicated that the employment status was not significantly different between genders (χ^2^(3) = 1.6, *p* = 0.7), but was significantly different between sexes (χ^2^(1) =29.4, *p* < 0.001). For the Employment * Sex interaction, chi-square tests revealed that males were significantly more likely than females to be employed or students rather than unemployed (OR = 4.64, *p* < 0.001) (see Tables 6, 9).

**Table 9:** Distribution of participants by employment status across the four gender categories.

Employment status	Male				Female				Total			
	Undif	Fem	Masc	Andr	Undif	Fem	Masc	Andr	Undif	Fem	Masc	Andr
Employed or student	**27** 22.5	**10** 8.3	**34** 28.3	**49** 40.8	**52** 26.5	**44** 22.4	**34** 17.3	**66** 33.7	**53** 27.0	**44** 22.4	**33** 16.9	**66** 33.7
Unemployed	**4** 28.6	**2** 14.3	**3** 21.4	**5** 35.7	**25** 23.6	**25** 23.6	**14** 13.2	**42** 39.6	**29** 27.4	**32** 30.2	**10** 9.4	**35** 33.0

Bold font numbers = *n*, normal numbers = %. Undif = undifferentiated; Fem = femininity; Masc = masculinity; Andr = androgynous.

#### Age

The differences between age groups (18–29, 30–39, 40–49, 50–59, and + 60), genders (4) and sexes (2) were also examined; the three-way loglinear analysis produced a final model that did not retain the three-way interaction (χ^2^(12) = 14.5, *p* = 0.27), but retained the two-way interactions (χ^2^(31) = 57.1, *p* = 0.003). Partial associations indicated that the age groups were significantly different between genders (χ^2^(12) = 22.8, *p* = 0.03), but not between sexes (χ^2^(4) = 1.7, *p* = 0.78).

For the Gender*Age interaction (see Tables 6, 10), chi-square tests revealed that individuals aged 18–29 were significantly more likely than those aged 40–49 to be categorized as Undifferentiated vs. Feminine (OR = 3.94, *p* = 0.001) and Undifferentiated vs. Androgynous (OR = 3.15, *p* = 0.004). In addition, those in the 18–29 age group were more likely than those aged 50–59 to be classified as Undifferentiated versus Androgynous (OR = 8.97, *p* = 0.002). Moreover, the 18–29-year-old group showed a marginally higher likelihood of identifying as Feminine compared to Androgynous than the 50–59 age group (OR = 4.38, *p* = 0.052). Similarly, they were more likely than those over 60 to identify as Undifferentiated vs. Androgynous (OR = 4.48, *p* = 0.054). These findings suggest modest but meaningful differences in gender identification patterns across age groups (see Table 11).

**Table 10:** Distribution of participants by age categories levels across four classifications.

Age	Male				Female				Total			
	Undif	Fem	Masc	Andr	Undif	Fem	Masc	Andr	Undif	Fem	Masc	Andr
18–29	**10** 28.6	**2** 5.7	**10** 28.6	**13** 37.1	**31** 38.3	**18** 22.2	**13** 16.0	**19** 23.5	**41** 35.3	**20** 17.2	**23** 19.9	**32** 27.6
30–39	**14** 23.0	**6** 9.8	**16** 26.2	**25** 41.0	**42** 30.7	**33** 24.0	**20** 14.6	**42** 30.7	**56** 28.2	**39** 19.7	**36** 18.2	**67** 33.9
40–49	**6** 23.1	**5** 19.2	**5** 19.2	**10** 36.5	**7** 12.3	**20** 35.1	**8** 14.0	**22** 38.6	**13** 15.7	**25** 30.1	**13** 15.7	**32** 38.6
50–59	**1** 20.0	**1** 20.0	**1** 20.0	**2** 40	**1** 5.3	**5** 26.3	**1** 5.3	**12** 63.1	**2** 8.3	**6** 25.0	**2** 8.3	**14** 58.3
+60	**1** 14.3	**1** 14.3	**4** 57.1	**1** 14.3	**1** 12.5	**0** 0	**1** 12.5	**6** 75.0	**2** 13.3	**1** 7.0	**5** 33.3	**7** 46.7

Bold font numbers = *n*, normal numbers = %. Undif = undifferentiated; Fem = femininity; Masc = masculinity; Andr = androgynous.

**Table 11:** Loglinear analysis effects break down for age categories vs. genders, for all participants.

Classifications	x^2^	*p*	Odds ratio
18–29 vs 30–39			
Undif vs. Masc	0.2	0.7	1.15
Undif vs. Fem	1.1	0.3	1.43
Undif vs. Andr	2.1	0.2	2.53
Masc vs. Fem	0.3	0.6	1.25
Masc vs. Andr	0.7	0.4	1.34
Fem vs. Andr	0.2	0.7	1.07
18–29 vs. 40–49			
Undif vs. Masc	1.5	0.7	1.78
Undif vs. Fem	10.3	0.001 ^a^	3.94
Undif vs. Andr	8.3	0.004 ^b^	3.15
Masc vs. Fem	3	0.08	2.21
Masc vs. Andr	1.8	0.2	1.77
Fem vs. Andr	0.3	0.6	0.8
18–29 vs. 50–59			
Undif vs. Masc	0.3	0.6	1.78
Undif vs. Fem	5.4	0.2	6.15
Undif vs. Andr	10.2	0.002 ^c^	8.97
Masc vs. Fem	2.2	0.1	3.45
Masc vs. Andr	4.7	0.3	5.03
Fem vs. Andr	0.4	0.5	1.46
18–29 vs. +60			
Undif vs. Masc	3.3	0.7	4.46
Undif vs. Fem	0	0.1	1.03
Undif vs. Andr	3.7	0.054	4.48
Masc vs. Fem	1.9	0.2	0.23
Masc vs. Andr	0	1	1.01
Fem vs. Andr	2.1	0.2	4.38

χ^2^ = Chi square. ^a^18-29 y.o more likely than 40–49 y.o to be undifferentiated vs feminine. ^b^18-29 y.o more likely than 40–49 y.o to be Undifferentiated vs Androgynous. ^c^18-29 y.o more likely than 50–59 y.o to be undifferentiated vs androgynous.

## Discussion

This study aimed to validate the expressivity (femininity) and instrumentality (masculinity) subscales of the culturally adapted short form of the Persian PAQ. The findings of this study support the validity of the Persian version of the PAQ, confirming its two-factor structure with instrumentality and expressivity, consistent with the original English version. The results also confirm the independence of these two factors, as indicated by a weak correlation. While most instrumentality-related items loaded on the instrumentality factor, the item “Active” did not, leading to the partial confirmation of the third hypothesis. Finally, the expressivity factor remained intact, with all related items loading as expected, confirming the fourth hypothesis. It also shows good internal consistency, and its test–retest reliability is moderate to high.

### Factorial structure of the Persian PAQ

The present findings are coherent with the factorial structure of the original short version of the PAQ, with factor extraction revealing a two-factor solution that corresponds to expressivity and instrumentality (Spence and Helmreich, 1978). The factor loadings for all participants indicated that the expressivity scale comprised the eight original expressivity items. Among the remaining eight items corresponding to the original instrumentality items, all items loaded on the instrumentality factor, but the item “Active” also loaded on the expressivity factor. By removing this item from the questionnaire, Cronbach’s alpha was reduced to 0.69, reflecting a decrease of 0.03. This indicates that the item ‘Active’ contributes to the variance shared by the other items and should be retained in the questionnaire. The first confirmatory factor analysis provided a good fit to the data (Yu, 2002). The second confirmatory factor analysis with added covariance did not show meaningful improvement, so the original structure was retained and included the item “Active” in the masculinity subscale.

The factorial structure of the present questionnaire is consistent with the original version of the PAQ, administered to high school and college students and their parents in the United States (Helmreich et al., 1981), to Lebanese undergraduate students (Spence and Helmreich, 1978) and the Canadian English-speaking population (Hill et al., 2000). It is also coherent with previous studies that used for Israeli students, Brazilian undergraduate students (Spence and Helmreich, 1978), the German version (Runge et al., 1981), the Chinese version (Moneta, 2010), and French-speaking females (K’delant and Gana, 2009) all of which demonstrated a two-factor solution. In contrast, these results are not consistent with the studies by Gaa et al. (1979), the British adaptation by McCreary and Steinberg (1992), and the French version by Hill et al. (2000), which demonstrated instability. Gaa et al. (1979) identified a four-factor solution in their analysis of 24 PAQ items, where two masculinity items ‘superior’ and ‘self-confident’ loaded more significantly on the fourth factor. Similarly, the study by McCreary and Steinberg (1992) analyzed 16 items of the PAQ among the British population and indicated a three-factor solution, with the ‘Active’ item loading onto the third factor. It is not clear why inconsistencies were observed in these versions, but the present Persian version aligns with most of the previous studies.

The internal consistency (*α* coefficients) of both factors is considered adequate, ranging between 0.69 and 0.73. The original PAQ by Spence and Helmreich (1978) achieved similar but higher reliability, with α coefficients of 0.82 for expressivity and 0.85 for instrumentality. In various adaptations of the PAQ, Cronbach’s α values were comparable to our study, ranging from 0.62 to 0.80. These include the study by Helmreich et al. (1981) across different age groups, the German translation by Runge et al. (1981), the British population study by McCreary and Steinberg (1992), studies across Canadian English-speaking and French-speaking populations by Hill et al. (2000), the Chinese translation by Moneta (2010), and French-speaking females (K’delant and Gana, 2009).

Besides, the intercorrelations between the two factors in this study are 0.30, indicating a weak correlation between expressivity and instrumentality. Similar weak correlations between expressivity and instrumentality were also reported in studies by Runge et al. (1981), Helmreich et al. (1981), Hill et al. (2000), Moneta (2010), and K’delant and Gana (2009). Together, the present and the previous results indicate that the relationship between expressivity and instrumentality is weak and that they are largely independent constructs.

### Test–retest reliability and stability of the responses

The instrumentality subscale of the PAQ showed high test–retest reliability (ICC = 0.92) over a one-month period, while the expressivity subscale showed moderate reliability (ICC = 0.69). The reliability difference for the two subscales could be due to the close association of the expressivity items with emotional attributes, which may exhibit greater variability over time. This remains speculative and future studies in larger samples are needed to confirm this finding. Nonetheless, the present findings indicate that the Persian version of PAQ is reliable. To our knowledge, no study reported the test–retest reliability of any version of the PAQ, so it is not possible to compare the present findings.

### Influence of sociodemographic factors on gender categories

As anticipated, the classification findings revealed that a higher number of males were categorized as Masculine rather than Feminine, whereas more females were categorized as Feminine rather than Masculine among sex-typed individuals. This aligns with the results obtained with the original version of PAQ and the German languages adaptation, which showed that males typically exhibit instrumental traits and are more frequently categorized as Masculine, while females tend to display expressive traits and are more often classified as Feminine (Runge et al., 1981; Spence and Helmreich, 1978). However, among Lebanese students, females were more often classified as feminine, while males were equally categorized as feminine and masculine (Spence and Helmreich, 1978). Moreover, female Israeli students were more often categorized as masculine rather than feminine, and Brazilian males were more frequently categorized as feminine rather than masculine (Spence and Helmreich, 1978). These discrepancies could be due to cultural differences and social norms, which is precisely what the PAQ intends to capture relative to gender, a collective construction of social roles.

Noteworthy, 61% of individuals were cross-typed while 39% were sex-typed in the present study. The high prevalence of cross-typed individuals, including androgynous and undifferentiated individuals, highlights that a large proportion of individuals do not strictly adhere to traditional gender roles and rather show a more balanced blend of expressivity and instrumentality. In this study, the proportion of individuals identifying as cross-typed is higher than what has been observed in the Lebanese, Israeli, and Brazilian populations (Spence and Helmreich, 1978). However, cross-typed individuals of both sexes were more frequently categorized as androgynous rather than undifferentiated in the present study and the study in Lebanese students (Spence and Helmreich, 1978). Besides, approximately 50 percent of participants were cross-typed in the German sample, with males more often categorized as undifferentiated and females as androgynous (Runge et al., 1981). In addition, among cross-typed Israeli and Brazilian students, males were more commonly categorized as undifferentiated, while females were predominantly classified as androgynous (Spence and Helmreich, 1978). This illustrates distinct patterns of gender expression across different linguistic and cultural contexts.

In the present study, genders were distributed differently between females and males. While gender roles evolve away from strictly sex-typed roles (Anbari and Fekri, 2021; Oláh et al., 2020), adherence to traditional feminine and masculine roles remains for both females and males. Accordingly, males clearly showed more adherence to traditional masculine roles compared with females, while females showed more adherence to traditional feminine roles compared with males (OR = 4.34, *p* < 0.001). However, some males were not categorized as masculine, and some females were not categorized as feminine, suggesting that these individuals are open to roles that are not typically associated with males or females, respectively.

In the context of the present study, these patterns can be attributed to traditional social and cultural expectations in Iran. From an early age, women receive mixed messages about gender roles: traditional socialization encourages adherence to feminine traits, while modern influences like feminist movements and social media emphasize qualities traditionally considered masculine, such as independence and assertiveness (Amini and Birjandi, 2012; Gheytanchi, 2015; Mortazavi and Poelker, 2017). According to the Ceasefire Centre for Civilian Rights, and Centre for Supporters of Human Rights, M.R.G.I. (2019), in a traditional society like Iran, males are still perceived as superior to women, and women are typically encouraged to adapt to conditions with greater flexibility. In addition, traditional gender roles provide more advantages to men (Balslev, 2019). Also, instrumentality is generally more valued than expressivity due to cultural and social norms, which favor men in many aspects of life, including employment and legal rights (Balslev, 2019; Mortazavi and Poelker, 2017). These social and cultural norms perpetuate traditional gender roles for both sexes. Moreover, the dual influences of tradition and modernity may lead to more undifferentiated gender identities among females and a heightened valuation of instrumentality, prompting males to either emphasize traditional masculinity, or adopt cross-gender traits.

The relationships between sociodemographic factors (age, education, and employment status), sex, and gender were examined in this study. Significant interactions were observed between sex and employment status, as well as between gender and age. Regarding the sex and employment, males were approximately 4.5 times more likely to be employed or students than females. This finding aligns with International Labour Organization (ILOSTAT) (2022) reports, which show that in Iran, the labor force participation rate among females and males in 2022 was 13.6 and 67.5%, respectively. This is consistent with the traditional Iranian society, in which females are more often at home than employed (Balslev, 2019).

Regarding age and gender, the distributions of genders were different between age groups. Individuals aged 18–29 were more likely to be categorized as undifferentiated rather than feminine or androgynous compared with individuals aged 40–49. Similarly, individuals aged 18–29 were more likely to be categorized as undifferentiated rather than androgynous compared with those aged 50–59. In addition, younger adults (18–29 years old) tended to be more likely than those over 60 to be categorized as undifferentiated rather than androgynous, although the difference was marginal (*p* = 0.54). This is consistent with previous findings by Shimonaka et al. (1997) showing that a higher number of adolescents aged 13–24 in the undifferentiated category compared with other categories. Considering that these studies were conducted over 25 years apart, these findings suggest that younger adults demonstrate less adherence to traditional gender roles compared to older adults. This may reflect psychological and affective development occurring over decades. Nonetheless, a previous study by Ghaffari’s (2019) on the Iranian population showed that the younger generation (Millennials) exhibits greater openness towards gender issues compared to the older generation (Generation X). Although this is the first study to assess gender roles with the PAQ in the Iranian population, future studies should clarify whether the differences between younger and older adults are related to age or cultural transformation.

Notwithstanding, age is likely a factor that influences gender. Fan and Marini (2000) stated that major life transitions, such as marriage, parenthood, and getting employment, can significantly impact gender roles. These milestones might contribute to shifts in how individuals perceive and engage with societal gender expectations (Fan and Marini, 2000). Gutmann (1977) proposed that individuals between 20 and 40 years old usually have a parental role and they are more sex-typed. In contrast, males between 40 and 60 years old tend to adopt traits considered more feminine, while women between 40- and 60-years old take on traits considered as more masculine, leading both genders towards the androgynous category. Similarly, around the midpoint of the life cycle, gender differences gradually diminish as people become more social and less ego-oriented (Hofstede et al., 2010). This shift leads to a decline in masculinity traits and a narrowing of the gap between men and women in these traits. Additionally, since women are no longer primarily responsible for child-rearing at this stage of life, fewer factors remain to differentiate them from men (Hofstede et al., 2010). These changes gradually eliminate gender role differences between men and women around age 45 (Hofstede et al., 2010).

The age-related changes in gender role classifications observed in this study generally align with the findings of Gutmann (1977) and Hofstede et al. (2010). However, the higher prevalence of the undifferentiated category among younger individuals (18–29 years old) in the present study, rather than the expected sex-typed classification, may be explained be several factors. Firstly, as mentioned earlier, Iranian women navigate dual roles, balancing traditional expectations and modern societal demands (Malekan, 2015), which may lead to roles overlap. Secondly, delayed economic and familial independence can postpone the adoption of traditional gender roles among men and women in Iran (Torabi and Shams Ghahfarokhi, 2021). Lastly, Iran’s diverse ethnic and cultural identities sometimes conflict with each other, leading to role confusion (Ahmady, 2023) and potentially delaying identity development in younger individuals. Accordingly, Gharaai et al. (2005) found that only a small percentage of 467 Iranian adolescents (16–18 y.o.) had reached the identity achievement stage. During the exploration phase of identity achievement, which involves trial and error, younger adults may exhibit various gender expressions and roles that do not necessarily align with traditional expectations (Erikson, 1959; Sinclair and Carlsson, 2013). In addition, the androgynous gender role is associated with identity achievement, while the undifferentiated gender role is associated with identity diffusion (Orlofsky et al., 1977). Since identity achievement is a long-term process, gender roles may remain undifferentiated until individuals achieve their identity, but this remains to be examined. Altogether, these findings suggest a developmental trajectory where individuals start with less defined gender roles and, as they age, may evolve towards embracing traits from their own gender or both genders. This progression highlights the fluid nature of gender as individuals move through different life stages.

### Application and implication

The strengths of the present study include a diverse sample ranging in age from 18 to 74 years old and representing different cities and ethnicities both inside and outside of Iran. Additionally, a meticulous linguistic adaptation was conducted by a team of experts, including a native translator and psychologists, to create a version closely resembling the original PAQ. Furthermore, the execution of a pilot study ensured cultural adaptation. Although the present results should be replicated in future studies using this version of the Persian PAQ, they show the validity, internal consistency, and test–retest reliability of the questionnaire.

### Limitations of the study

This study also presents some limitations. The online distribution of the questionnaire, leading to heterogeneity in the sample and biases in the diversity of social classes, education, ethnicity, age, and sex among participants. In addition, this questionnaire was distributed during the women’s protests in Iran in 2022, which could have influenced and introduced some bias in the gender classification. This should be taken into account in future studies.

### Future directions for PAQ research

This study provides a validated PAQ in Persian language to assess gender roles in adult Persian speakers. Research on the PAQ should be expanded, with future studies aiming for a balanced gender distribution across various ethnicities, age and social classes to enhance validation. In addition, conducting repeated cross-sectional studies could be beneficial for tracking the evolution of gender roles in Iran over time. Given that the PAQ has been validated in many languages and cultures, cross-cultural studies could also provide clearer insights into gender roles and stereotypes across different cultures and contexts. This questionnaire has broad applicability across various studies and research on gender-related phenomena. It holds particular significance for clinical research, such as those investigating the influence of gender roles on pain perception, as well as in the field of social science.

## Data Availability

The raw data supporting the conclusions of this article will be made available by the authors, without undue reservation.

## Supplementary material

The examiner and examinee Persian versions of the PAQ are available in the Supplementary material.
