# Supplementary material for: Assessing gender role attributes in native Persian speakers: translation, cultural adaptation, and validation of the Persian version of the personal attribute questionnaire
Source: Front Sociol. 2025 Mar 28;10:1535815. doi: 10.3389/fsoc.2025.1535815 (PMC11985821; doi:10.3389/fsoc.2025.1535815)
Supplement: Supplementary file 1 [file Presentation_1.pdf]

Persian Version of the Personal Attribute Questionnaire for Examinee

پرسشنامه صفات شخصی

دستور العمل:
موارد زیر درباره اینکه شما فکر می‌کنید چطور فردی هستید می‌باشد. هر مورد شامل یک جفت ویژگی با حروف الف، ب، ج، د، ه است. به عنوان مثال:

اصلا ذوق هنری ندارم الف ..... ب ...... ج .... د .... ه خیلی ذوق هنری دارم

هر جفت، ویژگی‌های متناقضی را توصیف می‌کند به این معنا که شما نمی‌توانید هم خیلی ذوق هنری داشته باشید و در عین حال اصلا ذوق هنری نداشته باشید.

مقیاس بین دو حد، با حروف مشخص می‌شود. شما باید حرفی را انتخاب کنید که توصیف می‌کند **شما** کجای مقیاس قرار دارید. به عنوان مثال اگر فکر می‌کنید اصلا ذوق هنری ندارید حرف "الف" را انتخاب کنید. اگر فکر می‌کنید نسبتا خوب هستید احتمالا حرف "د" را انتخاب می‌کنید. اگر فکر می‌کنید در حد متوسط هستید ممکن است "ج" را انتخاب کنید و به همین ترتیب.

١. اصلا مستقل نیستم	الف ..... ب ..... ج ..... د ..... ه	خیلی مستقل هستم
2. اصلا احساسی نیستم	الف ..... ب ..... ج ..... د ..... ه	خیلی احساسی هستم
3. خیلی منفعل هستم	الف ..... ب ..... ج ..... د ..... ه	خیلی فعال هستم
4. اصلا نمی‌توانم خودم را به طور کامل وقف دیگران کنم	الف ..... ب ..... ج ..... د ..... ه	می‌توانم خودم را کاملا وقف دیگران کنم
5. خیلی خشن هستم	الف ..... ب ..... ج ..... د ..... ه	خیلی ملایم هستم
6. اصلا کمک حال دیگران نیستم	الف ..... ب ..... ج ..... د ..... ه	خیلی کمک حال دیگران هستم
7. اصلا اهل رقابت نیستم	الف ..... ب ..... ج ..... د ..... ه	خیلی اهل رقابت هستم
8. اصلا مهربان نیستم	الف ..... ب ..... ج ..... د ..... ه	خیلی مهربان هستم
9. اصلا به احساسات دیگران آگاه نیستم	الف ..... ب ..... ج ..... د ..... ه	خیلی به احساسات دیگران آگاه هستم
10. می‌توانم به راحتی تصمیم بگیرم	الف ..... ب ..... ج ..... د ..... ه	تصمیم گیری برایم مشکل است
11. به راحتی منصرف می‌شوم	الف ..... ب ..... ج ..... د ..... ه	هیچگاه به راحتی منصرف نمی‌شوم
12. اصلا اعتماد به نفس ندارم	الف ..... ب ..... ج ..... د ..... ه	خیلی اعتماد به نفس دارم
13. خیلی احساس حقارت می‌کنم	الف ..... ب ..... ج ..... د ..... ه	خیلی احساس برتری می‌کنم
14. اصلا دیگران را درک نمی‌کنم	الف ..... ب ..... ج ..... د ..... ه	خیلی دیگران را درک می‌کنم
15. در روابط با دیگران خیلی سرد هستم	الف ..... ب ..... ج ..... د ..... ه	در روابط با دیگران خیلی گرم هستم
16. تحت فشار درهم می‌شکنم	الف ..... ب ..... ج ..... د ..... ه	تحت فشار خوب مقاومت می‌کنم

Persian Version of the Personal Attribute Questionnaire for Examiner

پرسشنامه صفات شخصی

دستور العمل:

موارد زیر درباره اینکه شما فکر می‌کنید چطور فردی هستید می‌باشد. هر مورد شامل یک جفت ویژگی با حروف الف، ب، ج، د، ه است. به عنوان مثال:

اصلا ذوق هنری ندارم الف ..... ب ...... ج .... د .... ه خیلی ذوق هنری دارم

هر جفت، ویژگی‌های متناقضی را توصیف می‌کند به این معنا که شما نمی‌توانید هم خیلی ذوق هنری داشته باشید و در عین حال اصلا ذوق هنری نداشته باشید.

مقیاس بین دو حد، با حروف مشخص می‌شود. شما باید حرفی را انتخاب کنید که توصیف می‌کند **شما** کجای مقیاس قرار دارید. به عنوان مثال اگر فکر می‌کنید اصلا ذوق هنری ندارید حرف "الف" را انتخاب کنید. اگر فکر می‌کنید نسبتا خوب هستید احتمالا حرف "د" را انتخاب می‌کنید. اگر فکر می‌کنید در حد متوسط هستید ممکن است "ج" را انتخاب کنید و به همین ترتیب.

م	۱. اصلا مستقل نیستم	الف ..... ب ..... ج ..... د ..... ه	خیلی مستقل هستم*
ز	2. اصلا احساسی نیستم	الف ..... ب ..... ج ..... د ..... ه	خیلی احساسی هستم*
م	3. خیلی منفعل هستم	الف ..... ب ..... ج ..... د ..... ه	خیلی فعال هستم*
ز	4. اصلا نمی‌توانم خودم را به طور کامل وقف دیگران کنم	الف ..... ب ..... ج ..... د ..... ه	می‌توانم خودم را کاملا وقف دیگران کنم*
ز	5. خیلی خشن هستم	الف ..... ب ..... ج ..... د ..... ه	خیلی ملایم هستم*
ز	6. اصلا کمک حال دیگران نیستم	الف ..... ب ..... ج ..... د ..... ه	خیلی کمک حال دیگران هستم*
م	7. اصلا اهل رقابت نیستم	الف ..... ب ..... ج ..... د ..... ه	خیلی اهل رقابت هستم*
ز	8. اصلا مهربان نیستم	الف ..... ب ..... ج ..... د ..... ه	خیلی مهربان هستم*
ز	9. اصلا به احساسات دیگران آگاه نیستم	الف ..... ب ..... ج ..... د ..... ه	خیلی به احساسات دیگران آگاه هستم*
م	10. می‌توانم به راحتی تصمیم بگیرم*	الف ..... ب ..... ج ..... د ..... ه	تصمیم گیری برایم مشکل است
م	11. به راحتی منصرف می‌شوم	الف ..... ب ..... ج ..... د ..... ه	هیچگاه به راحتی منصرف نمی‌شوم*
م	12. اصلا اعتماد به نفس ندارم	الف ..... ب ..... ج ..... د ..... ه	خیلی اعتماد به نفس دارم*
م	13. خیلی احساس حقارت می‌کنم	الف ..... ب ..... ج ..... د ..... ه	خیلی احساس برتری می‌کنم*
ز	14. اصلا دیگران را درک نمی‌کنم	الف ..... ب ..... ج ..... د ..... ه	خیلی دیگران را درک می‌کنم*
ز	15. در روابط با دیگران خیلی سرد هستم	الف ..... ب ..... ج ..... د ..... ه	در روابط با دیگران خیلی گرم هستم*
م	16. تحت فشار درهم می‌شکنم	الف ..... ب ..... ج ..... د ..... ه	تحت فشار خوب مقاومت می‌کنم*

مقیاسی که به هر مورد اختصاص داده شده است با ز (زنانگی) و م (مردانگی) مشخص شده است.
بخش‌های ستاره دار در مقیاس م حاکی از پاسخ مردانه افراطی و در مقیاس ز حاکی از پاسخ زنانه افراطی می‌باشد، که نمره 4 می‌گیرند. شدت بعدی نمره 3 گرفته و به همین ترتیب ادامه می‌یابد. (نمرات بین 0 تا 4 می‌باشند)
